# The dopamine D1 receptor agonist SKF81297 has dose-related effects on locomotor activity but is without effect in a CER trace conditioning procedure conducted with two versus four trials

**DOI:** 10.1016/j.lmot.2016.06.001

**Published:** 2016-08

**Authors:** M.A. Pezze, H.J. Marshall, H.J. Cassaday

**Affiliations:** School of Psychology, University of Nottingham, United Kingdom

**Keywords:** Dopamine D1, Trace conditioning, Contextual conditioning, Rat, SKF81297

## Abstract

In an appetitively motivated procedure, we have previously reported that systemic treatment with the dopamine (DA) D1 receptor agonist SKF81297 (0.4 and 0.8 mg/kg) depressed acquisition at a 2 s inter-stimulus-interval (ISI), suitable to detect trace conditioning impairment. However since DA is involved in reinforcement processes, the generality of effects across appetitively- and aversively-motivated trace conditioning procedures cannot be assumed. The present study tested the effects of SKF81297 (0.4 and 0.8 mg/kg) in an established conditioned emotional response (CER) procedure. Trace-dependent conditioning was clearly shown in two experiments: while conditioning was relatively strong at a 3-s ISI, it was attenuated at a 30-s ISI. This was shown after two (Experiment 1) or four (Experiment 2) conditioning trials conducted in – as far as possible – the same CER procedure. Contrary to prediction, in neither experiment was there any indication that trace conditioning was attenuated by treatment with 0.4 or 0.8 mg/kg SKF81297. In the same rats, locomotor activity was significantly enhanced at the 0.8 mg/kg dose of SKF81297. These results suggest that procedural details of the trace conditioning variant in use are an important determinant of the profile of dopaminergic modulation.

## 1 Introduction

Pavlovian trace conditioning procedures require the acquisition of an association between a conditioned stimulus (CS, e.g. noise) and an unconditioned stimulus (US, e.g. food or foot shock) despite the interposition of an interval of time between these events (Pavlov, 1927). The ability to condition to the trace of the CS when it is no longer present allows animals to form associations when events, which may nonetheless be causally-related, are separated in time. By definition, trace conditioning procedures test working memory defined as the capacity to maintain ‘on line’ transitory information (Goldman-Rakic, 1996). Such working memory is likely essential for associative processes in general, and in particular when a time interval must be bridged (Gilmartin, Balderston, & Helmstetter, 2014). Hence trace conditioning is widely used to investigate the neural substrates of this important aspect of working memory (Cassaday, Nelson, & Pezze, 2014).

The dominant paradigm used to study trace conditioning is eye blink conditioning in the rabbit which is known to rely on interactions between medial pre-frontal cortex (mPFC) and cerebellum (Kalmbach, Ohyama, Kreider, Riusech, & Mauk, 2009). Cerebellum may not be necessary for trace conditioning in other task variants and in any case it is important to test the generality of findings. Moreover, eye blink procedures typically use a very short trace or inter-stimulus-interval (ISI). We have therefore developed task variants suitable to examine trace conditioning over longer ISIs than the ms intervals typical of eye blink conditioning procedures.

In an appetitively motivated procedure, we have previously reported that systemic treatment with the dopamine (DA) D1 receptor agonist SKF81297 (0.4 and 0.8 mg/kg) depressed acquisition at a 2-s inter-stimulus-interval (ISI), suitable to detect trace conditioning impairment (Pezze, Marshall, & Cassaday, 2015). There was no effect on trace conditioning over the 10-s interval which was included to test for trace conditioning enhancement. This finding was counter to expectation in that systemic amphetamines reliably increase conditioning over a trace interval (Horsley and Cassaday, 2007, Norman and Cassaday, 2003). One possibility is that the motivation of the trace conditioning variant in use is an important determinant of the profile of dopaminergic modulation.

The conditioned emotional response (CER: noise → foot shock) procedures used in the present study are well established (Cassaday, Horsley, & Norman, 2005; Cassaday, Shilliam, & Marsden, 2001; Grimond-Billa, Norman, Bennett, & Cassaday, 2008; Horsley & Cassaday, 2007; Nelson, Thur, Spicer, Marsden, & Cassaday, 2011; Norman & Cassaday, 2003). These same procedures are sensitive to the effects of indirect DA agonists (systemically administered amphetamines: Horsley and Cassaday, 2007, Norman and Cassaday, 2003), as well as catecholaminergic depletion in nucleus accumbens (Nelson et al., 2011). Dopaminergic mechanisms are clearly involved in both appetitive (Dalley et al., 2002) and aversive conditioning (Feenstra, Vogel, Botterblom, Joosten, & de Bruin, 2001; Lauzon, Bechard, Ahmad, & Laviolette, 2013; Pezze & Feldon, 2004; Pezze, Bast, & Feldon, 2003). However, comparing across appetitive and aversive trace conditioning variants, there is evidence pointing to some differences in the underlying mechanisms (Cassaday et al., 2005).

Motivational valence inevitably affects other procedural parameters in that foot shock is more salient than food reward and salience is an important determinant of associative leaning. Many more learning trials are used in appetitive than is necessary or desirable in aversive procedures. In itself, the number of learning trials may also be a critical parameter in that a higher number of learning trials affords additional opportunity for consolidation, as well as an extended period of consolidation to the extent the duration of the conditioning session is increased along with the number of trials (Da Silva, Bast, & Morris, 2015; Domjan, 1980; Genoux, Haditsch, Knobloch, Michalon, Storm et al., 2002; McGaugh, Cahill, & Roozendaal, 1996). In CER procedures conducted at lower foot shock intensities, a greater number of pairings may result in a comparable level of conditioning to that typically seen after two pairings at higher foot shock intensity (Norman & Cassaday, 2003). Therefore, in the present study, we also used an increased numbers of pairings to promote consolidation, at a lower foot shock intensity to match – as far as possible – the baseline level of conditioned fear. Specifically, we compared the effects of SKF81297 (0.4 and 0.8 mg/kg) on trace conditioning conducted in a CER procedure using two (Experiment 1) or four (Experiment 2) pairings of a noise CS with foot shock, set at 1 mA (Experiment 1) or 0.5 mA (Experiment 2). In both experiments, we examined the effects of SKF81297 at 3-s and 30-s trace intervals which are suitable to test for impaired and enhanced trace CER conditioning, respectively. Thus we sought to examine the generality of the previous finding of impaired (short) trace conditioning obtained with SKF81297 in an appetitive procedure (Pezze, Marshall, & Cassaday, 2015) while including a longer trace suitable to test for trace conditioning enhancement (Horsley and Cassaday, 2007, Norman and Cassaday, 2003). Finally, the drug naïve (vehicle-injected) rats tested in Experiment 2 were subsequently used to examine the effects of the same doses of SKF81297 on locomotor activity (LMA) by way of positive control.

## 2 Materials and methods

### 2.1 Subjects

Seventy-two experimentally naïve adult male Wistar rats (Charles River, UK) were used for each experiment. They were caged in groups of four, in individually ventilated cages (IVCs) on a 12:12 h light/dark cycle with food and water ad libitum. After arrival, each rat was handled daily for one week and placed on water restriction 24 h prior to the start of each experiment. The mean start weight was 220 g (range 196–239 g) in Experiment 1 and 212 g (range 191–246 g) in Experiment 2. All procedures were conducted in accordance with the UK Animal Scientific Procedures Act 1986, Project Licence numbers: PPL 40/3163 (Experiment 1) and PPL 40/3716 (Experiment 2).

### 2.2 Drug treatments

SKF81297 (Tocris, UK) was dissolved in saline (0.9% NaCl) to provide an injectable volume of 1 ml/kg at doses of 0.4 mg/kg and 0.8 mg/kg. Drug doses were based on previous studies run in our laboratory (Nelson, Thur, & Cassaday, 2012; Pezze, Marshall, & Cassaday, 2015). In both experiments, vehicle (saline) or SKF81297 (0.4 mg/kg or 0.8 mg/kg) were injected subcutaneously (s.c.) 15 min prior to the conditioning stage of the trace conditioning procedure. The same treatments were further examined in LMA.

### 2.3 Trace conditioning apparatus

Both experiments were conducted using 6 duplicate fully automated conditioning chambers (Cambridge Cognition, Cambridge, UK), housed within sound-attenuating cases fitted with ventilation fans. The conditioning boxes were made of plain steel (25 cm × 25 cm × 22 cm high) with a Plexiglas door (19 cm × 27 cm) inset at the front. A waterspout was mounted on one wall, 5 cm above the floor and connected to a lickometer supplied by a pump. The number of licks made was registered by a break in the photo beam within the waterspout, which also triggered the delivery of water (0.05 ml per lick). The waterspout was illuminated when water was available. A loudspeaker set in the roof of each conditioning box produced the CS (tone), which consisted of a 5-s mixed frequency noise set at 85 dB (including background); this was presented at either 3-s or 30-s trace intervals before the US (foot shock). The foot shock of 1 s duration set at 1 mA (Experiment 1) or 0.5 mA intensity (Experiment 2) provided the US. This shock was delivered through the grid floor (steel bars 1 cm apart) by a constant current shock generator (pulsed voltage: output square wave 10 ms on, 80 ms off, 370 V peak under no load conditions; MISAC Systems, Newbury, UK). Three wall-mounted stimulus lights and the house light were set to flash on (0.5 s) and off (0.5 s) for the duration of the conditioning session, thus providing an experimental background stimulus. Stimulus control and data collection were performed by an Acorn Archimedes RISC computer programme in Basic with additional interfacing using an Arachnid extension (Cambridge Cognition).

### 2.4 LMA apparatus

Twelve clear Perspex chambers (39.5 cm long × 23.5 cm wide × 24.5 cm deep) with metal grid lids were used (Photobeam Activity System, San Diego Instruments, USA). The chambers were surrounded by frames containing two levels of photobeams as described previously (Jones, Brown, Auer, & Fone, 2011; Pezze, McGarrity, Mason, Fone, & Bast, 2014; Pezze, Marshall, Fone, & Cassaday, 2015). Two consecutive breaks of adjacent beams within the lower level of photobeams generated a locomotor count. The apparatus was situated in a dimly lit (50-70 lx) room. To start a session, rats were placed into the centre of the chamber. Total locomotor counts were recorded for each consecutive 10-min epoch, for 30 min pre-treatment and 60 min post-treatment.

### 2.5 Behavioural conditioning procedure

Water restriction was introduced 1 day prior to shaping. The rats received 1 h ad libitum access to water in their home cages after each of the procedural stages described below. This home cage access was in addition to any water drunk in the conditioning boxes (available from the apparatus waterspout on all days of the procedure apart from the conditioning day). Therefore the rats were trained, conditioned and tested in counterbalanced groups of six after 20–23 h of water restriction.

#### 2.5.1 Pre-conditioning to establish baseline lick response

In order to initiate licking behaviour, rats were shaped over two days. On the first day, rats were placed in the conditioning boxes in pairs (with their cage mates), where they were given one or more 15-min sessions to learn how to drink from the illuminated waterspout. On the second day of shaping, they were individually allocated to a conditioning box to ensure that all rats were independently drinking freely. Thereafter, the rats were assigned to their individual boxes for the duration of the experiment (counterbalanced by experimental group). No data were recorded.

Five days of pre-training followed, in which rats drank for 15 min each day (timed from first lick). The waterspout was illuminated throughout, but no other stimuli were present in this phase. The latency to first lick was measured as an indicator of habituation to the experimental context. In addition the total number of licks was also analysed to assess any pre-existing differences in baseline drinking (prior to conditioning).

#### 2.5.2 Conditioning with foot shock

The waterspouts were not illuminated and no water was available during the conditioning session. The US (foot shock) was delivered following the termination of the CS (tone) at either 3-s or 30-s trace intervals. There were two pairings of CS and US in Experiment 1 and four pairings of CS and US in Experiment 2. The flashing light experimental background stimulus was presented for the duration of the conditioning session. In Experiment 1, the first pairing of CS and US was presented after 5 min had elapsed, and the second pairing was 5 min after the first, followed by a further 5 min spent in the apparatus. The same procedure was used in Experiment 2 with two additional CS and US pairings (totalling four). The first pairing was presented after 5 min had elapsed, with the following three pairings presented at 5-min intervals followed by a further 5 min spent in the apparatus. In the absence of licking, no behavioural measures were recorded.

#### 2.5.3 Re-shaping after foot shock

In order to re-establish drinking behaviour after conditioning, rats were re-shaped the following day. This followed the same procedure used in the pre-conditioning, in which rats drank for 15 min (timed from first lick). Conditioning to the box context was measured as the latency to first lick, as well as the profile of drinking over the 15 min session.

#### 2.5.4 Test

Conditioned suppression to the experimental stimuli was tested on two consecutive days following re-shaping. Rats were placed in the conditioning boxes and presented with the CS (tone) on day 1 and the background stimulus (light) on day 2. Water was available throughout the test and the waterspout was illuminated. After the rats had made 50 licks, the stimulus tone (day 1) or light (day 2) was presented for 15 min. The time taken to make the first 50 licks in the absence of any stimulus (the A period) provided a measure of any individual variation in baseline lick responding. This was compared with the time taken to complete 50 licks following stimulus onset (the B period) in a suppression ratio (A/(A + B)) to determine the level of conditioning to either stimulus, adjusted for any individual baseline variation. The profile of drinking over the 15 min session provided an additional measure of conditioned suppression.

### 2.6 LMA procedure

This was tested two replications, each of 12 rats from the vehicle-injected group of Experiment 2. Due to the malfunction of one of the activity chambers, data from two of the available rats was not collected. One day before the drug tests, each rat was placed in a test chamber for 30 min to habituate it to the box. The habituation activity data were also used to match the rats’ allocation to drug groups (so that there were no differences in baseline activity). On the following day, rats were replaced in the same test chamber for 30 min to achieve further habituation and to facilitate the detection of any SKF81297-induced LMA. Rats were then subcutaneously injected with saline, or 0.4 or 0.8 mg/kg of SKF81297 and immediately replaced in their allocated activity boxes for 60 min.

### 2.7 Experimental design and analysis

**Fig. 1:**
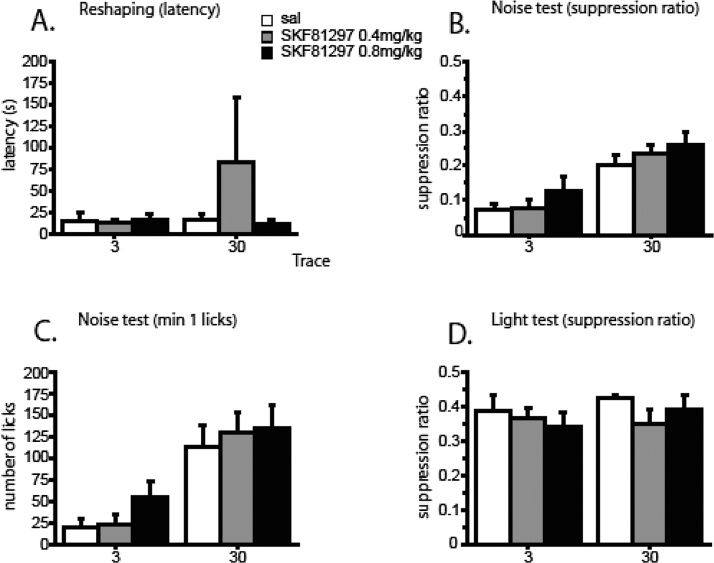
Effects on conditioning of the D1 receptor agonist SKF81297 at 0 (white columns), 0.4 (grey columns), and 0.8 (black columns) mg/kg in Experiment 1. The error bars show the standard error of the mean (n = 9-12/group). Tests were conducted drug-free after prior conditioning under drug conducted with two CS (noise) → US (1 mA shock) pairings, presented using either a 3-s or 30-s trace interval. A. Suppression to the experimental chambers: the level of contextual conditioning is expressed as mean latency to make the first lick (s). B. Conditioned suppression to the noise CS expressed as the mean suppression ratio. C. Conditioned suppression to the noise CS measured as the number of licks in the first min of test presentation. D. Conditioned suppression to the light background expressed as the mean suppression ratio.

**Fig. 2:**
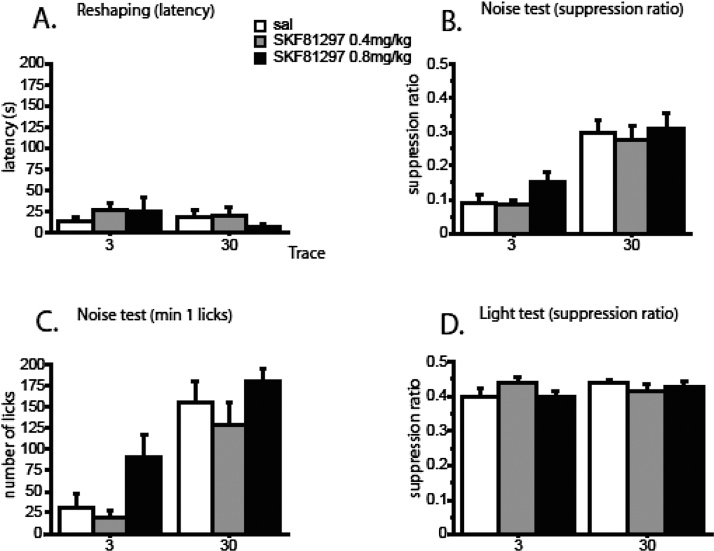
Effects on conditioning of the D1 receptor agonist SKF81297 at 0 (white columns), 0.4 (grey columns), and 0.8 (black columns) mg/kg in Experiment 2. The error bars show the standard error of the mean (n = 10-12/group). Tests were conducted drug-free after prior conditioning under drug conducted with four CS (noise) → US (0.5 mA shock) pairings, presented using either a 3-s or 30-s trace interval. A. Suppression to the experimental chambers: the level of contextual conditioning is expressed as mean latency to make the first lick (s). B. Conditioned suppression to the noise CS expressed as mean suppression ratio. C. Conditioned suppression to the noise CS measured as the number of licks in the first min of test presentation. D. Conditioned suppression to the light background expressed as the mean suppression ratio.

In both experiments, there were six experimental groups run in a 2 × 3 independent factorial design. The between subject factors were trace at levels 3 s or 30 s and drug at doses saline or SKF81297 (0.4 mg/kg or 0.8 mg/kg). The dependent variables to check for differences by experimental condition-to-be were pre-conditioning drink latencies and the number of licks made during the 15-min pre-conditioning session. Contextual conditioning to the box cues was measured by the reshaping drink latencies and the number of licks made during the 15-min reshaping session. On each of the test days, the level of conditioning to the CS or the experimental background stimulus was measured by the suppression ratios and the number of licks made during the 15-min test session. The licks analyses were run in a mixed design with the repeated measures factor of min which had 15 levels. In the case of significant interactions with min, follow up ANOVA was restricted to the first 60 s of drinking (min 1) which typically shows the closest correspondence with the pattern of effects shown on the suppression ratios at relatively low levels of suppression. Figs. 1 and 2 show suppression ratios and min 1 licks for the noise tests of both experiments, to allow direct comparison. The locomotor effects of SKF81297 were tested in mixed design with drug as the between-subjects factor and 10 min blocks of activity counts as the within-subjects factor. Follow up ANOVAs by individual block were conducted to explore the interaction. Where required, post hoc tests were performed by Fisher’s LSD test.

In Experiment 1, the data of ten rats were excluded from the light test analyses, because of equipment failure on the light test day. This left a final sample size of 62 (n = 9-12/cell). Three rats’ data were excluded in Experiment 2, due to equipment failure on the noise test day. This left a final sample size of 69 (n = 10-12/cell). Two rats’ data were missing from the LMA analyses, leaving a final sample size of 22 (n = 7-8/cell).

## 3 Results

### 3.1 Experiments 1 with 2 conditioning trials

#### 3.1.1 Pre-conditioning—baseline licking

There were no effects of drug, F(2,66) = 1.968, p = 0.148, or trace condition-to-be, F < 1, on the latency to start drinking on the final pre-conditioning day. This confirms that the rats’ drinking was well-matched prior to any conditioning. An ANOVA performed on the number of licks made over the 15-min session showed the expected main effect of min, F(14,924) = 109.126, p < 0.0001. However, the decline in drinking over the course of the session was uninfluenced by drug, F(28,924) = 1.175, p = 0.244, or trace condition-to-be, F(14,924) = 1.189, p = 0.278 (data not shown).

#### 3.1.2 Reshaping—contextual conditioning

On the reshaping day there was no statistical evidence for any effect of prior drug or trace condition on the level of drinking at the start of the session (Fig. 1A). There was no difference in the latency to first drink by drug or trace, nor any interaction between these factors, all Fs <1. There was a main effect of min on the number of licks made over the 15 min session, F(14,924) = 72.639, p < 0.0001. However, there was no effect of drug by min, F(28,924) = 0.633, p = 0.931, or trace by min, F(14,924) = 1.225, p = 0.251 (Table 1A). There was some suggestion that drug treatment at conditioning had some influence on contextual conditioning in that there was a min × drug × trace interaction, F(28,924) = 1.613, p = 0.024, but there was no effect by drug on the min 1 measure, both Fs < 1.

#### 3.1.3 CER test—noise CS

There was a main effect of trace on the suppression ratio measure, F(1,66) = 35.099, p < 0.0001, because licking behaviour in rats conditioned with a 30-s trace was less suppressed than licking behaviour in rats conditioned with a 3-s trace interval (Fig. 1B). There was no effect of drug, F(2,66) = 2.228, p = 0.116, or of the trace × drug interaction, F < 1. There was a main effect of min on the number of licks, F(14,924) = 21.758, p < 0.0001, and, more importantly, an interaction between min and trace, F(14,924) = 7.648, p < 0.0001. Drug was significant only by min in the linear trend, F(2,66) = 3.646, p = 0.031. Once rats were drinking freely, those which had been treated with 0.8 mg/kg SKF81297 showed the steepest drop in drinking over the session (Table 1B). However, there was no effect by drug on the min 1 measure, maximum, F(2,66) = 1.166, p = 0.318. Just as would be expected, there was a main effect of trace, F(1,66) = 33.724, p < 0.001 (Fig. 1C).

#### 3.1.4 CER test—flashing lights background

There was no difference in the suppression ratio scores by trace or drug condition, and there was no interaction between drug and trace, all Fs <1 (Fig. 1D). On the licks measure there was a main effect of min, F(14,784) = 55.579, p < 0.0001, but drinking was independent of prior trace, F(14,784) = 0.697, p = 0.778, and drug treatment, F(28,784) = 0.999, p = 0.468 (Table 1C).

### 3.2 Experiment 2 with 4 conditioning trials

#### 3.2.1 Pre-conditioning—baseline licking

There were no effects of drug, F(2,63) = 0.996, p = 0.375, or trace condition-to-be, F(1, 63) = 1.868, p = 0.177, on latencies to start drinking on the final pre-conditioning day. This shows that the rats’ drinking was well-matched prior to any conditioning. An ANOVA performed on the number of licks made over the 15-min session showed the expected main effect of min, because the rats drank more at the beginning of the session, F(14, 882) = 103.964, p < 0.001. However, drinking over the session was not moderated by drug, F(28, 882) = 0.748, p = 0.825, or trace condition-to-be, F(14,882) = 1.251, p = 0.232 (data not shown).

#### 3.2.2 Reshaping—contextual conditioning

On the day following conditioning there was no evidence of any effect of drug or trace condition on drinking at the start of the session (Fig. 2A). There was no difference in the drink latencies by drug, F(2,63) = 0.392, p = 0.678, or trace, F(1,63) = 0.732, p = 0.395, and there was no interaction between drug and trace, F(2,63) = 0.725, p = 0.488. The number of licks declined over the course of the 15-min session. Statistically, there was a main effect of min, F(14,882) = 77.385, p < 0.001. However, the pattern of drinking over time was not influenced by drug, F(28,882) = 0.761, p = 0.810, or trace, F(14,882) = 1.396, p = 0.148 (Table 2A).

#### 3.2.3 CER test—noise CS

There was a main effect of trace on the suppression ratio, F(1,63) = 43.625, p < 0.001. As expected, rats conditioned with a 30 s trace interval were less suppressed than rats conditioned with a 3 s trace interval (Fig. 2B). However, there was no effect of drug, either overall, F(2,63) = 1.198, p = 0.309, or in interaction with trace, F(2,63) = 2.275, p = 0.111. An ANOVA done to analyse the licks measure showed a main effect of min, F(14,882) = 34.495, p < 0.001. Animals initially drank less, reflecting fear conditioning to the noise CS, but drinking later peaked and then dropped over the course of the 15 min session. There was both an overall effect of trace, F(1,63) = 7.228, p = 0.009, and an interaction between trace and min, F(14,882) = 6.779, p < 0.001, as the difference between the 3-s and 30-s conditioned groups dropped over the course of the session (Table 2B). Moreover, the interaction between drug and min was significant in the linear trend, F(2,63) = 4.252, p = 0.019, as was the three-way interaction, F(2,63) = 3.532, p = 0.035. ANOVA restricted to min 1 drinking showed a main effect of drug, F(2,63) = 4.832, p = 0.011, as well as the expected effect of trace, F(1,63) = 39.843, p = 0.001(Fig. 2C). The main effect of drug seen in min 1 arose because rats previously conditioned under 0.8 mg/kg SKF81297 drank more than those conditioned under 0.4 mg/kg SKF, p = 0.003. However neither of the groups conditioned under SKF81297 was significantly different from saline, smallest p = 0.064, for the 0.8 mg/kg group.

#### 3.2.4 CER test—flashing lights background

There was no difference in the suppression ratios by trace, F(1,63) = 1.258, p = 0.266, or the drug condition, F(2,63) = 0.496, p = 0.611, and there was no interaction between drug and trace, F(2,63) = 1.713, p = 0.188 (Fig. 2D). On the licks measure, ANOVA showed the expected main effect of min, F(14,882) = 104.715, p < 0.001. There were no interactions between min and trace, F(14,882) = 1.144, p = 0.315, or between min and drug, F(28,882) = 0.821, p = 0.731. The three-way interaction was significant, F(28,882) = 1.646, p = 0.019. Inspection of the means (shown in Table 2C) suggests that this may have arisen because of a relatively steeper fall off in drinking in rats conditioned under SKF81297 at the 3-s ISI (compared to their respective saline controls), combined with less of a drop in rats conditioned under SKF81297 at the 30-s ISI (compared to their respective saline controls). However, the three-way interaction was significant in the quadratic rather than the linear trend, F(2.63) = 4.112, p = 0.021, and ANOVA restricted to min 1 showed no effect of drug, both Fs < 1.

### 3.3 Locomotor activity

**Fig. 3:**
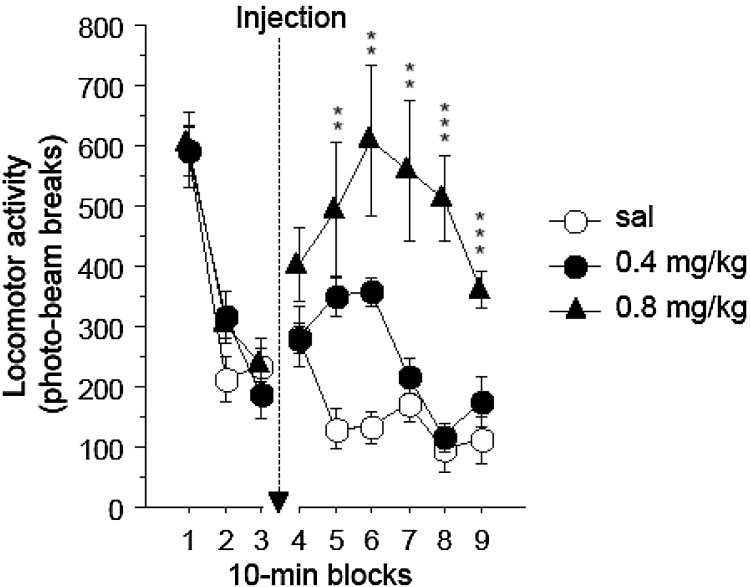
Effect of systemic injections of SKF81297 on locomotor activity monitored for 60 min post injection (in blocks 5–9). For comparison, blocks 1–3 show habituation to the activity chambers over 30 min prior to injection. The error bars show the standard error of the mean (n = 7–8 rats per group). Asterisks indicate a significant difference as compared to saline: **p < 0.01; ***p < 0.001.

The groups were initially well-matched in that in the 30 min preceding any injection there were no differences by drug condition-to-be, maximum F(4,38) = 2.033, p = 0.109, for the interaction with blocks. Then over the next 60 min, systemic administration of SKF81297 increased activity at both doses compared to saline starting 10 min after injection and lasting for the further 50 min test duration (Fig. 3). There was an overall effect of drug, F(2,19) = 9.899, p = 0.001, because rats treated with 0.8 mg/kg SKF81297 were more active than those treated with 0.4 mg/kg (p = 0.006) or saline (p < 0.001). The activity of rats treated with 0.4 mg/kg SKF81297 was not significantly different from that seen in the saline-injected controls (p = 0.251). There was an interaction between blocks and drug, F(10,95) = 5.310, p < 0.001. Follow up analyses showed that this arose because although there was no difference by drug dose in the first 10-min block after injection, F(2,19) = 2.043, p = 0.157, there was an effect of drug in all subsequent 10 min blocks, minimum F(2,19) = 5.832, p = 0.011. Further post hocs confirmed that in blocks 5–9, there was a significant difference between the saline and the 0.8 mg/kg SKF81297-treated rats (p = 0.003 in block 5 and p < 0.001 in block 9). The difference between the saline and 0.4 mg/kg SKF81297-treated rats did not reach significance in any block, though it was marginal in block 5 (p=0.059) and block 6 (p = 0.070).

## 4 Discussion

Trace-dependent conditioning was clearly shown in the present experiments in that while conditioning was relatively strong at the 3-s ISI, it was attenuated at the 30-s ISI. This was shown after two (Experiment 1) or four (Experiment 2) conditioning trials conducted in what was otherwise – as far as possible – the same CER procedure. Contrary to prediction, in neither experiment was there any indication that trace conditioning was attenuated by treatment with 0.4 or 0.8 mg/kg SKF81297.

The expected effect of increased LMA under SKF81297 provides a positive control for the effectiveness of this particular batch of drug (Pezze, Marshall, & Fone, 2015b). Neither was SKF81297 completely without effect in the trace conditioning procedure in that there was some evidence for reduced conditioning (measured as relatively increased min 1 licking) under 0.8 mg/kg SKF81297, seen in Experiment 2 which was conducted with an increased number of conditioning trials. There were also some indications that treatment with SKF81297 moderated (the expression of) contextual conditioning as measured in the pattern of drinking seen in the experimental boxes at reshaping in Experiment 1 or during the light test in Experiment 2. This latter effect is broadly consistent with the results seen after localised micro-infusion of SKF81297 in the anterior cingulate part of the mPFC. However, with the more localised administration and the standard two conditioning trials there was evidence for overall increased conditioning to the experimental background (Pezze, Marshall, Domonkos, & Cassaday, 2016). In the present study, the observed effect was not so systematic. Moreover, there was no effect on suppression to the light background in either direction in Experiment 1 of the present study which used 2 conditioning trials.

With respect to the primary objective of the study, to assess effects on trace conditioning, there was no evidence for any reduction in trace conditioning after D1 receptor stimulation in the CER procedure used in the present study. This result is inconsistent with the effects of 0.4 and 0.8 mg/kg SKF81297 in appetitive trace conditioning (Pezze, Marshall, & Cassaday, 2015). This discrepancy raises the possibility that appetitive versus aversive motivation of the trace conditioning task, in other words the nature of the US, is an important determinant of the susceptibility of trace conditioning to dopaminergic modulation (Cassaday et al., 2005). For example, particular combinations of stimuli do not start out equally effective in conditioning, to the extent that some CS-US relationships are learned more easily than others (Garcia & Koelling, 1966; Shapiro, Jacobs, & LoLordo, 1980). However, with an appropriate adjustment to the ISI, trace-dependent conditioning is robustly demonstrated across a variety of conditioning preparations motivated by different appetitive and aversive USs (Cassaday et al., 2005; Horsley & Cassaday, 2007; Kalmbach et al., 2009; Norman & Cassaday, 2003; Pezze, Marshall, & Cassaday, 2015). Therefore, with the right behavioural parameters, the effects of drugs and lesions on trace conditioning might in principle be expected to hold across different procedural variants. However, given the role of dopaminergic systems in the reinforcement mechanisms underlying conditioning (Bromberg-Martin, Matsumoto & Hikosaka, 2010; Dalley et al., 2002; Feenstra et al., 2001; Horvitz, 2000; Iordanova, 2009; Pezze & Feldon, 2004; Salamone, 1994), such stimulus-reinforcer interactions are likely to influence the susceptibility of associative learning to dopaminergic treatments, as has been found to be the case in latent inhibition procedures (Cassaday & Moran, 2010; Moser, Lister, Hitchcock, & Moran, 2000).

Ideally, we would have compared the effects of SKF81297 (and other DA agonists) using appetitive and aversive USs under directly comparable training conditions. Inevitably, over and above differences which may be attributable to task motivation per se, the task motivation in use has secondary effects on other aspects of the procedure, such as, for example, the number of conditioning trials. CER trace procedures only require two conditioning trials and – as a legal requirement – the number of foot shock US deliveries should be the minimum required to support the required level of associative learning. Appetitive conditioning takes place over many trials, up to over 100 in total (Cassaday et al., 2005; Pezze, Marshall, & Cassaday, 2015), and we found that reliable demonstration of within day learning requires the use of 30 conditioning trials (Pezze, Marshall, & Cassaday, 2015). Therefore the number of conditioning trials cannot in practice be equated between appetitive and aversive procedures. However, trace CER procedures have been conducted using 4 conditioning trials and a lower (0.5 mA) foot shock intensity (Norman & Cassaday, 2003) and – adopting these procedures – the effects of SKF81297 were further examined in Experiment 2 of the present study. However, within the constraints of the UK Animal Scientific Procedures Act 1986 Project Licence (PPL 40/3716), we were unable to establish any effect of SKF81297 on trace fear conditioning using the maximum number of conditioning trials permitted (4 trials conducted at half the standard foot shock intensity). Thus the lack of effect of SKF81297 was confirmed (in so far as we were able to test this) independent of the number and intensity of foot shocks.

Similarly, it is not in practice possible to match temporal aspects of appetitive and aversively motivated procedures because the ISIs suitable for optimal associative learning are different to begin with. We have routinely used a maximum ISI of 10 s in appetitive trace conditioning (Cassaday et al., 2005; Pezze, Marshall, & Cassaday, 2015) compared with 30 s which has been found suitable to detect enhanced trace conditioning in the aversive procedure (Horsley and Cassaday, 2007, Nayak and Cassaday, 2003, Norman and Cassaday, 2003). The typical inter-trial-intervals are also rather longer for foot shock procedures. The fact that foot shocks are not given in quick succession allows the animals time to recover, and the required number of conditioning trials can be accommodated within a 15–25 min conditioning session (Cassaday et al., 2005, Norman and Cassaday, 2003).

The response requirements of appetitively- and aversively-motivated procedures are also different. However, direct effects on response rate are excluded by the lack of any response requirement during conditioning when drug treatments are administered and the use of drug-free tests in the CER procedures of the present study. Moreover, drug effects on response rates are controlled for in appetitive trace conditioning procedures (Cassaday et al., 2005; Pezze, Marshall, & Cassaday, 2015).

Because other DA agonists have previously been investigated using the same CER procedures, direct comparisons can be drawn with previously published studies. In the present study, we saw no evidence for enhanced trace conditioning as has been demonstrated after treatment with amphetamines in the CER procedure and at the 30-s trace interval used in the present experiments (Horsley and Cassaday, 2007, Norman and Cassaday, 2003). Albeit using a longer 60-s trace interval, the DA D4 agonist PD 168,077 was similarly without effect in a CER procedure (Nayak & Cassaday, 2003). Taken together with the findings of the present experiments, this lack of effect with more selective receptor agents would seem to suggest that the overall profile of actions at different DA receptor sub-types and/or noradrenergically-mediated effects may mediate increased trace conditioning with an aversive US. Nonetheless procedural differences beyond motivation are likely to be critical. In a conditioned freezing procedure (the retention of) trace conditioning has been found to be impaired by DA D1 blockade in mPFC (Runyan & Dash, 2004). Moreover, in contrast to the present study, Runyan and Dash (2004) used a procedure designed to minimize contextual associations. We have routinely used an experimental background stimulus, which most likely increases the salience of context as well as providing a measure of contextual conditioning which can be tested in the same way as the CS (Horsley and Cassaday, 2007, Nayak and Cassaday, 2003, Norman and Cassaday, 2003).

We find that effects observed in appetitively-motivated trace conditioning do not simply reproduce in an aversively-motivated CER procedure, despite the fact that the CER procedure in use has previously shown sensitivity to dopaminergic manipulations and within the present experiments there were some signs of drug effects in measures of contextual conditioning, albeit not particularly systematic. The particular sensitivity of the appetitive task variant is consistent with the dominant view that DA mainly signals reward value (Schultz, 2010). Even within this motivational system, the incentive value of the US can affect the profile of sensitivity to different DA receptor agents (Olarte-Sánchez, Valencia-Torres, Cassaday, Bradshaw, & Szabadi, 2013). Such differences will inevitably influence the dose-response function seen with individual compounds. Thus, we cannot exclude the possibility that different doses of SKF81297 would have been more effective in the present procedure, particularly given the results earlier obtained with mPFC infusions of SKF81297 (Pezze, Marshall, & Cassaday, 2015) and SCH23390 (Runyan & Dash, 2004).

We acknowledge that differences in the neural substrates of trace conditioning may relate to a multitude of procedural differences in addition to that of the reinforcers in use. Moreover, it is important to stress that such differences do not preclude the existence of neural substrates which mediate aspects of associative learning, such as trace conditioning, across paradigms. Nonetheless, discrepancies of the kind discussed above constrain the delineation of the neuropharmacological substrates of different facets of associative learning irrespective of task variant. Moreover, at the behavioural level the aim has been to establish general theoretical models of learning. The present data may be taken to suggest that different variants of the relevant learning theories may be needed to account for behaviour in tasks which are motivated aversively vs. appetitively, perhaps by formal inclusion of reinforcement sensitivity theory (Gray and McNaughton, 2000, Gray, 1982).

Finally, given the imperative to refine experimental procedures (Russell & Burch, 1959, Chapter 7), it is increasingly important to acknowledge that while appetitive procedures may be better from an animal welfare perspective, other inevitable differences – likely motivational, but perhaps due to other methodological differences – can change the conclusions to be drawn.

## Conflicts of interest

None.

## Figures and Tables

**Table 1:** Licking behaviour over the 15 min sessions conducted at (A) reshaping, (B) the noise test and (C) the light test subsequent to treatment with the D1 receptor agonist SKF81297 (SKF) at 0, 0.4 and 0.8 mg/kg in Experiment 1. The table shows the mean number of licks made in each min ± the standard error of the mean. Tests were conducted drug-free after prior conditioning under SKF conducted with two CS (noise) → US (1 mA shock) pairings, presented using either a 3 s or 30 s trace interval.

A. Reshaping						
Trace	3 s			30 s		
Drug	Sal	0.4 SKF	0.8 SKF	Sal	0.4 SKF	0.8 SKF
Min						
1	202.750 ± 28.239	226.333 ± 24.863	211.000 ± 14.960	227.333 ± 16.627	208.500 ± 28.332	219.833 ± 20.827
2	191.667 ± 20.791	207.000 ± 23.583	185.083 ± 17.194	169.333 ± 20.409	147.583 ± 19.085	163.667 ± 13.017
3	163.083 ± 24.823	163.250 ± 14.691	155.333 ± 18.246	165.417 ± 21.496	167.667 ± 19.248	165.750 ± 16.945
4	144.833 ± 25.087	129.250 ± 24.038	126.083 ± 21.704	154.917 ± 25.805	138.500 ± 19.927	141.833 ± 23.522
5	121.333 ± 26.173	192.000 ± 19.897	116.333 ± 23.667	150.667 ± 26.655	127.333 ± 18.162	138.333 ± 13.796
6	108.167 ± 27.579	137.583 ± 23.341	74.083 ± 20.020	166.750 ± 20.307	101.083 ± 19.558	148.000 ± 25.575
7	132.667 ± 26.060	48.083 ± 12.852	121.500 ± 27.200	111.000 ± 25.445	120.000 ± 21.759	86.833 ± 16.878
8	73.000 ± 23.584	54.167 ± 17.281	34.083 ± 12.277	83.667 ± 31.328	39.000 ± 15.216	87.000 ± 23.460
9	66.917 ± 22.640	51.333 ± 18.054	47.500 ± 22.368	54.083 ± 20.973	63.833 ± 22.967	70.167 ± 28.729
10	54.333 ± 21.091	32.750 ± 16.474	76.000 ± 23.739	64.083 ± 25.222	47.500 ± 13.996	40.667 ± 13.293
11	62.000 ± 18.182	39.167 ± 13.439	12.333 ± 6.922	58.083 ± 23.147	21.583 ± 15.558	64.333 ± 23.410
12	31.250 ± 21.437	26.667 ± 11.683	35.500 ± 15.895	30.500 ± 12.321	37.500 ± 17.502	54.833 ± 26.808
13	39.250 ± 15.893	54.500 ± 25.917	26.000 ± 14.404	4.000 ± 3.645	23.917 ± 9.672	9.167 ± 5.396
14	43.250 ± 20.096	12.417 ± 8.196	24.250 ± 12.437	16.500 ± 9.902	29.250 ± 12.464	27.917 ± 9.460
15	42.417 ± 18.947	21.083 ± 15.0200	6.917 ± 6.826	12.500 ± 10.826	36.500 ± 8.609	51.917 ± 24.811

B. Noise test						
Trace	3 s			30 s		
Drug	Sal	0.4 SKF	0.8 SKF	Sal	0.4 SKF	0.8 SKF
Min						
1	20.667 ± 10.357	22.833 ± 12.567	56.583 ± 17.261	113.000 ± 25.138	130.333 ± 22.662	135.917 ± 24.542
2	62.250 ± 26.232	70.083 ± 30.160	131.417 ± 27.623	161.000 ± 27.908	177.500 ± 8.593	207.167 ± 18.356
3	93.167 ± 29.525	95.250 ± 29.600	150.000 ± 27.924	125.000 ± 19.271	159.583 ± 19.064	201.333 ± 19.806
4	122.333 ± 30.279	101.500 ± 29.968	159.250 ± 28.873	167.167 ± 21.338	172.083 ± 13.284	144.917 ± 17.890
5	111.250 ± 23.638	120.667 ± 32.568	177.750 ± 29.103	143.167 ± 26.263	114.417 ± 16.963	132.333 ± 27.089
6	136.167 ± 22.669	113.000 ± 26.581	126.917 ± 20.100	85.750 ± 20.902	119.667 ± 21.371	134.833 ± 23.732
7	111.167 ± 31.156	136.000 ± 25.675	124.667 ± 33.330	118.417 ± 21.351	55.583 ± 15.986	78.917 ± 21.097
8	98.833 ± 28.623	124.750 ± 30.004	67.917 ± 23.152	124.167 ± 32.047	73.167 ± 19.793	103.250 ± 23.897
9	117.667 ± 27.052	119.917 ± 26.733	116.917 ± 23.382	83.500 ± 24.872	105.500 ± 22.889	58.250 ± 22.364
10	98.083 ± 27.136	127.167 ± 22.489	112.583 ± 26.639	70.417 ± 23.219	66.750 ± 13.694	51.417 ± 14.176
11	63.667 ± 19.800	78.917 ± 28.806	46.083 ± 12.348	54.000 ± 20.892	29.167 ± 10.551	51.750 ± 21.620
12	47.667 ± 21.513	89.500 ± 18.017	59.167 ± 18.739	33.750 ± 13.778	42.750 ± 13.979	17.500 ± 8.957
13	70.000 ± 21.302	93.250 ± 20.240	62.000 ± 17.447	39.167 ± 19.459	25.750 ± 12.260	6.833 ± 4.846
14	33.000 ± 13.375	45.083 ± 19.685	24.917 ± 12.321	51.250 ± 18.344	23.083 ± 11.206	27.167 ± 10.740
15	41.250 ± 18.808	25.250 ± 13.811	18.750 ± 17.236	39.667 ± 17.751	35.333 ± 11.543	23.500 ± 11.020

C. Light test						
Trace	3 s			30 s		
Drug	Sal	0.4 SKF	0.8 SKF	Sal	0.4 SKF	0.8 SKF
Min						
1	194.222 ± 36.321	219.400 ± 30.768	202.000 ± 29.447	222.600 ± 20.647	169.167 ± 28.812	212.545 ± 30.884
2	188.000 ± 33.042	231.400 ± 20.749	179.900 ± 29.531	209.600 ± 14.811	192.833 ± 20.935	185.636 ± 25.594
3	163.222 ± 32.100	233.700 ± 24.281	172.400 ± 23.865	185.200 ± 22.719	165.833 ± 19.908	166.364 ± 18.960
4	164.333 ± 33.067	196.100 ± 21.714	162.600 ± 29.847	157.700 ± 20.087	138.500 ± 27.366	146.727 ± 32.314
5	120.667 ± 33.384	131.500 ± 31.680	146.900 ± 22.000	153.300 ± 29.088	161.000 ± 18.989	153.091 ± 21.918
6	107.000 ± 33.872	118.100 ± 27.533	123.900 ± 23.624	150.800 ± 26.368	124.000 ± 16.642	58.455 ± 18.216
7	132.778 ± 33.168	117.300 ± 17.989	77.100 ± 31.154	148.200 ± 20.672	47.167 ± 18.253	67.455 ± 24.309
8	126.000 ± 34.137	120.900 ± 26.409	75.300 ± 23.321	63.400 ± 28.637	85.833 ± 25.531	102.091 ± 26.361
9	57.333 ± 24.927	49.400 ± 14.741	90.500 ± 25.976	68.100 ± 21.420	72.250 ± 23.573	91.545 ± 28.232
10	47.111 ± 27.504	42.000 ± 19.693	47.600 ± 21.662	47.800 ± 23.056	39.833 ± 11.458	20.182 ± 9.571
11	52.889 ± 20.402	25.400 ± 11.294	34.200 ± 16.817	40.100 ± 26.116	29.833 ± 11.971	37.909 ± 20.212
12	60.667 ± 19.857	50.400 ± 27.414	13.900 ± 8.093	24.000 ± 16.277	17.750 ± 12.389	10.455 ± 8.388
13	50.444 ± 25.868	18.600 ± 11.768	31.000 ± 14.844	43.200 ± 13.670	29.750 ± 13.005	37.636 ± 21.417
14	27.333 ± 15.408	25.100 ± 17.642	28.000 ± 9.843	30.400 ± 12.317	17.667 ± 12.579	9.545 ± 5.808
15	104.667 ± 34.721	31.800 ± 18.587	59.100 ± 15.959	61.400 ± 22.952	31.667 ± 14.159	42.364 ± 18.037

**Table 2:** Licking behaviour over the 15 min sessions conducted at (A) reshaping, (B) the noise test and (C) the light test subsequent to treatment with the D1 receptor agonist SKF81297 (SKF) at 0, 0.4 and 0.8 mg/kg in Experiment 2. The table shows the mean number of licks made in each min ± the standard error of the mean. Tests were conducted drug-free after prior conditioning under drug conducted with four CS (noise) → US (0.5 mA shock) pairings, presented using either a 3 s or 30 s trace interval.

A. Reshaping						
Trace	3 s			30 s		
Drug	Sal	0.4 SKF	0.8 SKF	Sal	0.4 SKF	0.8 SKF
Min						
1	219.400 ± 23.910	252.417 ± 13.516	232.000 ± 17.219	235.273 ± 15.936	200.333 ± 11.481	217.917 ± 17.219
2	192.500 ± 21.749	194.250 ± 18.844	182.000 ± 20.006	180.545 ± 14.008	158.750 ± 16.068	194.333 ± 20.006
3	126.300 ± 26.559	185.167 ± 21.536	168.000 ± 19.952	170.364 ± 14.154	165.083 ± 18.528	171.417 ± 19.952
4	175.500 ± 21.127	129.000 ± 16.616	172.333 ± 24.838	197.091 ± 14.247	165.667 ± 22.494	145.083 ± 24.838
5	124.000 ± 19.938	119.750 ± 19.353	157.333 ± 24.264	166.909 ± 29.597	137.083 ± 24.548	151.750 ± 24.264
6	133.100 ± 22.213	163.917 ± 32.946	145.667 ± 24.398	116.545 ± 22.739	97.500 ± 23.501	116.083 ± 24.398
7	104.200 ± 30.831	77.833 ± 13.092	110.333 ± 21.970	91.727 ± 26.255	48.417 ± 16.750	87.500 ± 21.970
8	74.500 ± 20.281	83.250 ± 18.257	85.417 ± 20.083	121.000 ± 22.614	89.250 ± 23.736	81.333 ± 20.083
9	68.400 ± 30.518	84.333 ± 25.449	44.250 ± 12.035	88.182 ± 17.317	60.417 ± 24.646	66.000 ± 12.035
10	62.500 ± 25.892	47.000 ± 12.012	71.500 ± 26.719	63.455 ± 18.024	28.083 ± 10.413	36.000 ± 26.719
11	40.000 ± 23.171	45.417 ± 15.109	31.417 ± 10.607	62.273 ± 22.757	33.833 ± 23.514	55.250 ± 10.607
12	62.500 ± 26.705	39.333 ± 18.945	49.250 ± 14.171	31.182 ± 22.946	13.250 ± 12.533	58.917 ± 14.171
13	27.400 ± 12.548	32.500 ± 21.329	15.000 ± 7.638	67.000 ± 26.248	29.000 ± 12.484	36.083 ± 7.638
14	22.500 ± 11.592	28.917 ± 10.344	13.583 ± 6.689	16.364 ± 8.561	35.500 ± 12.203	20.167 ± 9.689
15	14.700 ± 7.727	14.167 ± 7.569	15.167 ± 11.999	14.818 ± 8.396	38.417 ± 22.009	31.167 ± 11.999

B. Noise test						
Trace	3 s			30 s		
Drug	Sal	0.4 SKF	0.8 SKF	Sal	0.4 SKF	0.8 SKF
Min						
1	30.900 ± 22.637	18.583 ± 19.325	91.500 ± 26.411	155.182 ± 22.637	128.917 ± 19.325	180.333 ± 13.888
2	64.200 ± 16.446	123.333 ± 15.752	131.583 ± 31.135	204.000 ± 16.446	178.750 ± 15.742	213.000 ± 15.521
3	79.800 ± 20.543	146.583 ± 22.970	148.250 ± 22.569	189.455 ± 20.543	211.000 ± 22.970	183.833 ± 19.078
4	114.100 ± 23.953	155.167 ± 25.985	157.167 ± 28.449	191.455 ± 23.953	156.917 ± 25.985	226.667 ± 14.498
5	112.300 ± 19.552	114.250 ± 28.386	162.750 ± 23.111	159.545 ± 19.552	178.333 ± 28.386	151.333 ± 15.539
6	85.900 ± 31.776	121.000 ± 15.562	155.417 ± 27.845	130.545 ± 31.776	136.167 ± 15.562	124.417 ± 22.413
7	90.000 ± 30.834	125.250 ± 24.151	151.750 ± 29.676	91.727 ± 30.834	93.417 ± 24.151	99.083 ± 16.868
8	97.800 ± 24.401	118.750 ± 28.424	122.333 ± 29.080	140.818 ± 24.401	93.833 ± 28.424	62.500 ± 17.506
9	110.100 ± 30.670	50.750 ± 24.243	98.250 ± 17.777	142.091 ± 30.670	89.833 ± 24.243	78.167 ± 20.008
10	69.600 ± 9.663	63.167 ± 19.572	101.333 ± 31.702	63.636 ± 9.663	81.750 ± 19.572	48.000 ± 22.186
11	49.000 ± 17.780	52.083 ± 14.917	73.750 ± 19.429	67.727 ± 17.780	53.417 ± 14.917	55.750 ± 14.402
12	112.500 ± 11.275	66.833 ± 10.637	37.667 ± 9.700	21.000 ± 11.275	60.833 ± 10.637	63.000 ± 24.069
13	61.200 ± 9.478	29.667 ± 16.531	18.500 ± 6.336	42.364 ± 9.478	46.833 ± 16.531	27.083 ± 9.407
14	46.800 ± 15.200	13.917 ± 14.663	23.750 ± 9.634	34.727 ± 45.200	37.667 ± 14.663	31.250 ± 12.688
15	38.200 ± 19.402	12.167 ± 5.699	46.500 ± 20.493	22.182 ± 19.402	25.667 ± 5.699	28.250 ± 17.092

C. Light test						
Trace	3 s			30 s		
Drug	Sal	0.4 SKF	0.8 SKF	Sal	0.4 SKF	0.8 SKF
Min						
1	213.200 ± 22.637	241.833 ± 19.325	237.417 ± 19.107	246.091 ± 9.051	235.833 ± 14.389	232.833 ± 15.119
2	215.700 ± 16.446	203.500 ± 15.742	202.583 ± 18.311	208.091 ± 14.130	193.250 ± 19.958	233.250 ± 12.792
3	194.600 ± 20.543	182.667 ± 22.970	178.750 ± 24.148	237.909 ± 18.239	202.667 ± 13.380	232.417 ± 14.651
4	152.900 ± 23.953	138.883 ± 25.985	196.250 ± 21.783	195.364 ± 28.447	182.833 ± 31.407	176.083 ± 24.513
5	121.400 ± 19.552	129.167 ± 28.386	173.083 ± 23.324	196.727 ± 23.586	183.333 ± 28.771	172.417 ± 19.967
6	106.000 ± 31.776	143.917 ± 15.562	112.833 ± 16.875	159.727 ± 19.284	146.417 ± 22.854	115.000 ± 23.029
7	80.000 ± 30.834	114.167 ± 24.151	191.083 ± 25.129	168.364 ± 28.907	104.333 ± 23.833	111.000 ± 18.209
8	61.000 ± 24.401	53.520 ± 28.424	87.917 ± 25.031	63.818 ± 24.287	119.833 ± 27.705	65.667 ± 20.280
9	56.700 ± 30.670	83.167 ± 24.243	70.333 ± 19.932	64.364 ± 22.517	120.667 ± 24.911	81.750 ± 16.272
10	27.400 ± 9.663	56.583 ± 19.572	31.667 ± 11.91	76.000 ± 18.875	57.917 ± 21.354	76.917 ± 25.204
11	31.800 ± 17.780	33.500 ± 14.917	72.083 ± 16.419	32.182 ± 11.243	25.500 ± 9.953	39.833 ± 16.540
12	20.500 ± 11.275	29.883 ± 10.637	13.083 ± 7.586	2.091 ± 2.091	29.333 ± 12.306	41.000 ± 11.101
13	19.500 ± 9.478	35.417 ± 16.531	35.750 ± 16.417	19.636 ± 12.228	23.750 ± 10.798	27.000 ± 12.498
14	15.200 ± 15.200	45.667 ± 14.663	20.083 ± 13.376	14.545 ± 8.545	48.083 ± 16.037	38.167 ± 9.898
15	56.400 ± 19.402	8.500 ± 5.699	3.667 ± 2.802	2.727 ± 2.000	57.667 ± 25.104	47.667 ± 18.428
